# Treatment of excessive gingival display using conventional esthetic crown lengthening versus computer guided esthetic crown lengthening: (a randomized clinical trial)

**DOI:** 10.1186/s12903-024-04080-5

**Published:** 2024-03-09

**Authors:** Eman Borham, Hala Ahmed Abuel-Ela, Islam Shawky Mohamed, Yasmine Ahmed Fouad

**Affiliations:** 1 Assistant Lecturer of Oral Medicine and Periodontology, Faculty of Dentistry, Misr International University, Cairo, Egypt; 2 Professor of Oral Medicine, Periodontology and Oral Diagnosis, Faculty of Dentistry, Ain Shams University and Misr International University, Cairo, Egypt; 3 Lecturer of Oral and Maxillofacial Radiology, Faculty of Dentistry, Misr International University, Cairo, Egypt; 4 Lecturer of Oral Medicine, Periodontology and Oral Diagnosis, Faculty of Dentistry, Ain Shams University and Misr International University, Cairo, Egypt; 5 Omarat Misr ELTameer Sheraton Heliopolis, 16 Abd ELHameed Badawy, Cairo, Egypt

**Keywords:** Altered passive eruption, Dual surgical guide, Esthetic crown lengthening, Excessive gingival display, Guided surgery, Gummy smile

## Abstract

**Background:**

Surgical guides have been proposed in an attempt to reach more predictable outcomes for esthetic crown lengthening. The objective of the present study was to evaluate the effectiveness of esthetic crown lengthening using 3D-printed surgical guides in the management of excessive gingival display due to altered passive eruption type 1B.

**Materials and methods:**

Sixteen patients diagnosed with altered passive eruption type 1B, were divided into two groups. In the control group, the procedure was carried out conventionally, and in the study group, a dual surgical guide was used. The parameters of wound healing (swelling, color, probing depth, bleeding index, and plaque index), pain scores, gingival margin stability, and operating time were assessed at 1 week, 2 weeks, 3 months, and 6 months postoperatively.

**Results:**

There was no statistically significant difference in terms of wound healing, pain scores, and gingival margin stability between both groups at different time intervals (*P* = 1), however, there was a statistical difference between both groups in terms of operating time with the study group being significantly lower (*P* < 0.001).

**Conclusion:**

Digitally assisted esthetic crown lengthening helps shorten the operating time and reduces the possibility of human errors during the measurements. This will be useful in helping practitioners achieve better results.

**Practical implications:**

The conventional method remains to be the gold standard. However, shorter operating time and lower margins for errors will help reduce costs as the chair side time is reduced as well as the possibility for a second surgery is lower. This will improve patient satisfaction as well.

**Supplementary Information:**

The online version contains supplementary material available at 10.1186/s12903-024-04080-5.

## Clinical Trial Registry date

23/08/2022.

**Clinical Trial Registry Number: NCT**05512312.

## Introduction

Excessive gingival display (EGD) or gummy smile is identified as displaying more than 2 mm of gingiva during maximum smiling. According to esthetics standards it is considered to be unesthetic, requiring attention [1]. The origins of EGD could be skeletal as in cases of vertical maxillary excess, muscular as in cases of short/hypertonic upper lips, or dentogingival abnormalities like in cases of altered passive eruption (APE) or a combination of these causes [2]. The most common etiology is APE, where the gingival margin is located at a more coronal location as a result of the failure of the passive stage of tooth eruption resulting in a short clinical crown. APE is morphologically classified into 1 A: Osseous crest apical to CEJ, adequate amount of keratinized gingiva. 1B: Osseous crest at CEJ, adequate amount of keratinized gingiva. 2 A: Osseous crest apical to CEJ, inadequate amount of keratinized gingiva. 2B: Osseous crest at CEJ, inadequate amount of keratinized gingiva [3]. . In cases of APE, the ideal treatment would be crown lengthening to increase the length of the clinical crown and simultaneously reduce the EGD. The type of crown lengthening procedure to be executed if the only reason for the procedure is to reduce the EGD, and no restorations are to be used in adjunction depends on the width of the keratinized gingiva and the relationship of the alveolar crest to the cementoenamel junction (CEJ) [4].

The procedure to be discussed in this study is gingivectomy with ostecotomy in the treatment of APE type 1B. This procedure however can be unstable and unpredictable; in some cases, the gingiva might regrow and relapse either completely or incompletely, or in other incidences, the gingiva may recede beyond the levels to where it was adjusted [5, 6]. Thus, different modalities have been proposed in the literature in an attempt to reach more predictable and stable outcomes. These modalities included periodontal measurements, the use of surgical gauges, hand-fabricated stents, and digitally guided surgical stents [7–9]. While each of these methods proved to improve the outcomes and stability, digitally guided surgical stents are theoretically the most accurate and efficient as it is guided by the pre-surgical bone levels measured on the cone beam computed tomography (CBCT), thus eliminating the chance of human errors during taking measurements.

In 2020, the Standard Tessellation Language (STL) files (from intraoral scanning or scanning of impressions) were overlapped with Digital Imaging and Communications in Medicine (DICOM) files (from CBCT) to determine the amount of soft and hard tissue that needs to be removed. This alignment increases the accuracy of the guide to be printed as it facilitates the visualization of the distance from the CEJ to the bone crest and from the gingival margin to the CEJ in millimeters. In addition, it aids in the diagnosis of APE and is thus considered a valuable tool [10]. 3D technology has been implemented in printing surgical guides, decreasing the operating time and lowering the complication rate which results in increased patient acceptance and satisfaction [11].

There is no consensus that digitally guided Esthetic Crown Lengthening (ECL) is the ideal modality for the most esthetic and stable results. Thus, this study was carried out to compare the stability of gingival margin levels, pain levels, wound healing, and operating time after digitally guided ECL versus conventional ECL, in an attempt to shed light on the importance and convenience of digital dentistry in esthetic periodontal surgery.

## Methods

### Study design

**Fig. 1:**
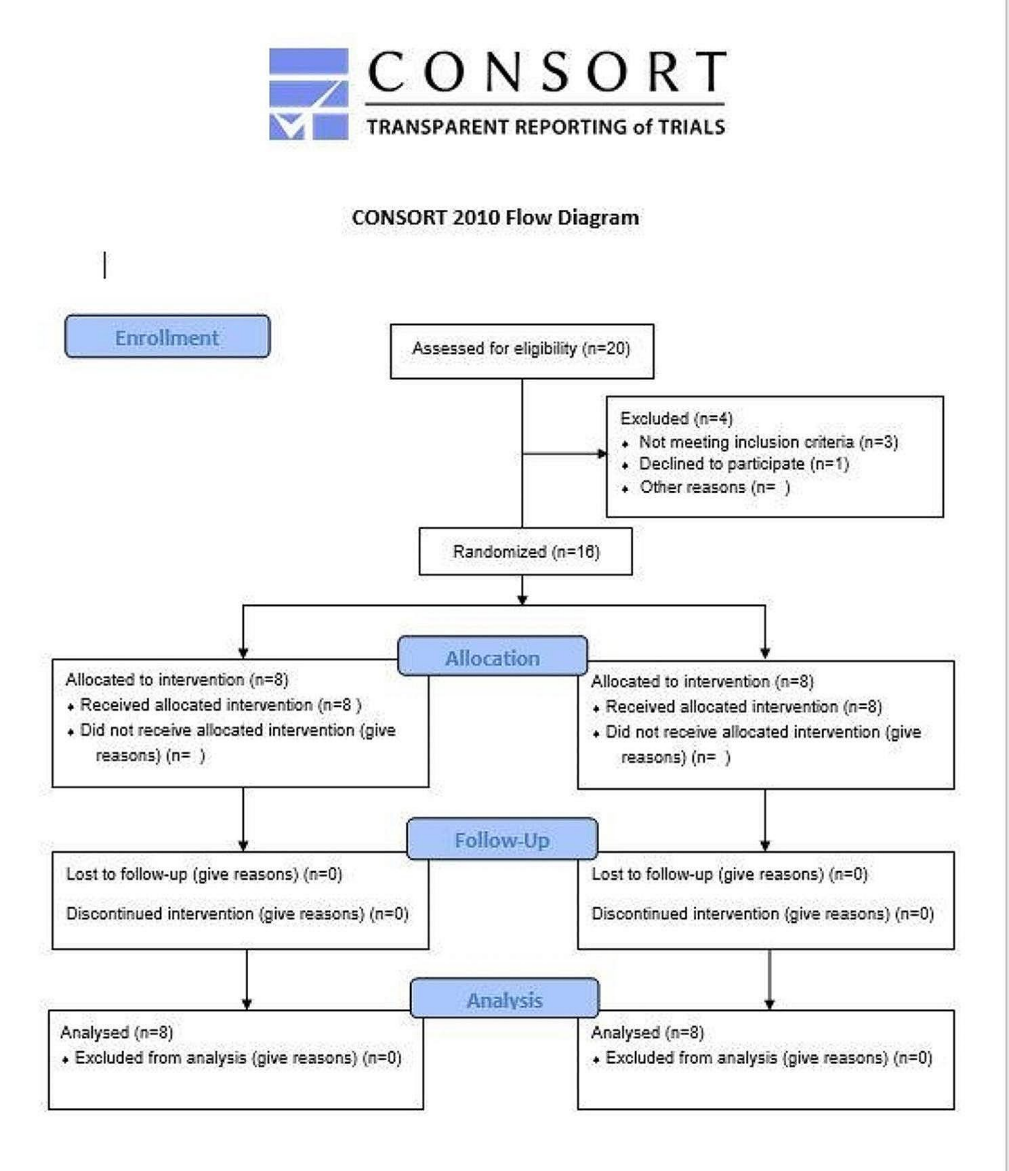
CONSORT flow chart

This study was a randomized controlled clinical trial. The study protocol was approved by the Research Ethics Committee with registration number (FDASU-RecIM11200). This clinical trial was conducted by CONSORT guidelines. All patients were committed to the treatment protocol throughout the six months of the study period with no dropouts as demonstrated in the study flow diagram provided in Fig. (1).

### Study sample

Sixteen patients participated in this randomized clinical trial. The patients were divided randomly into two groups, eight per group using a predetermined computer-generated randomization list^1^ Allocation concealment was ensured using a sealed coded envelope containing the treatment of the participant. Each patient was randomly assigned to (control group): conventional ECL. (study group): digitally guided CL. The outcome assessor (Y.A) and the statistician were blinded in contrast to the skilled surgeon (E.B) and patients as the performed interventions were different. The purpose of the study was explained to all patients and an informed consent was signed before the conduction of the study. The inclusion criteria were as follows: Gummy smile due to APE type 1B, age range of 20–40 years, systemically (medically) free, patients with thick gingival phenotype, and good compliance with the plaque control instructions. Patients were excluded if they were smokers, pregnant or lactating, had teeth with compromised periodontium, using medications that affect periodontal wound healing, or had periodontal surgery in the esthetic zone in the past 6 months.

### Preoperative phase

A full-mouth periodontal chart was recorded for all patients that included plaque index (PI), bleeding index (BI), probing depth (PD), and clinical attachment level (CAL) recorded from the gingival margin to the base of the pocket at six sites per tooth (mesiobuccal, distobuccal, mid-buccal, mesiolingual, disto-lingual, and mid-lingual). Clinical parameters were recorded using a graduated UNC-15 periodontal probe.^2^ All the patients enrolled were periodontally healthy. An individually customized index with guiding grooves was fabricated from putty condensation cure silicone and served as a reference point for gingival margin levels that were used later during the follow-ups (1 week, 2 weeks, 3 months, and 6 months postoperatively) where the parameters were measured using the same technique.

**Fig. 2:**
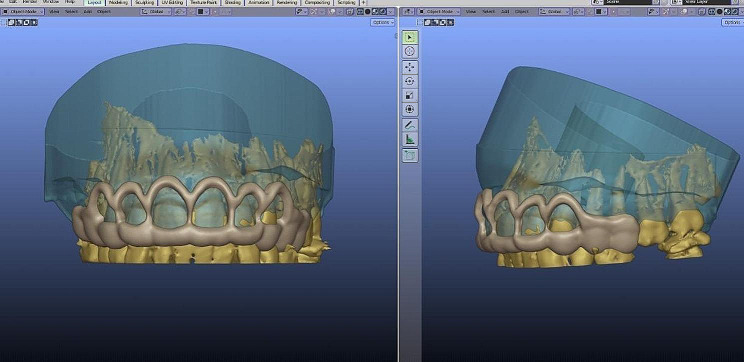
Overlapping of the DICOM and STL files and guide design

**Fig. 3:**
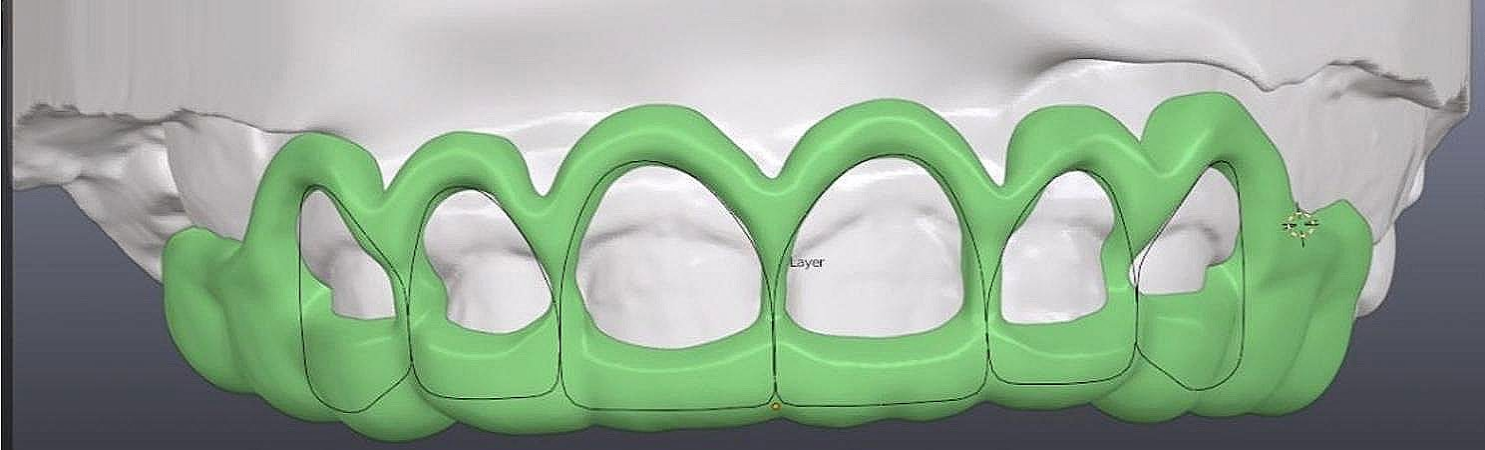
Final surgical guide design

**Fig. 4:**
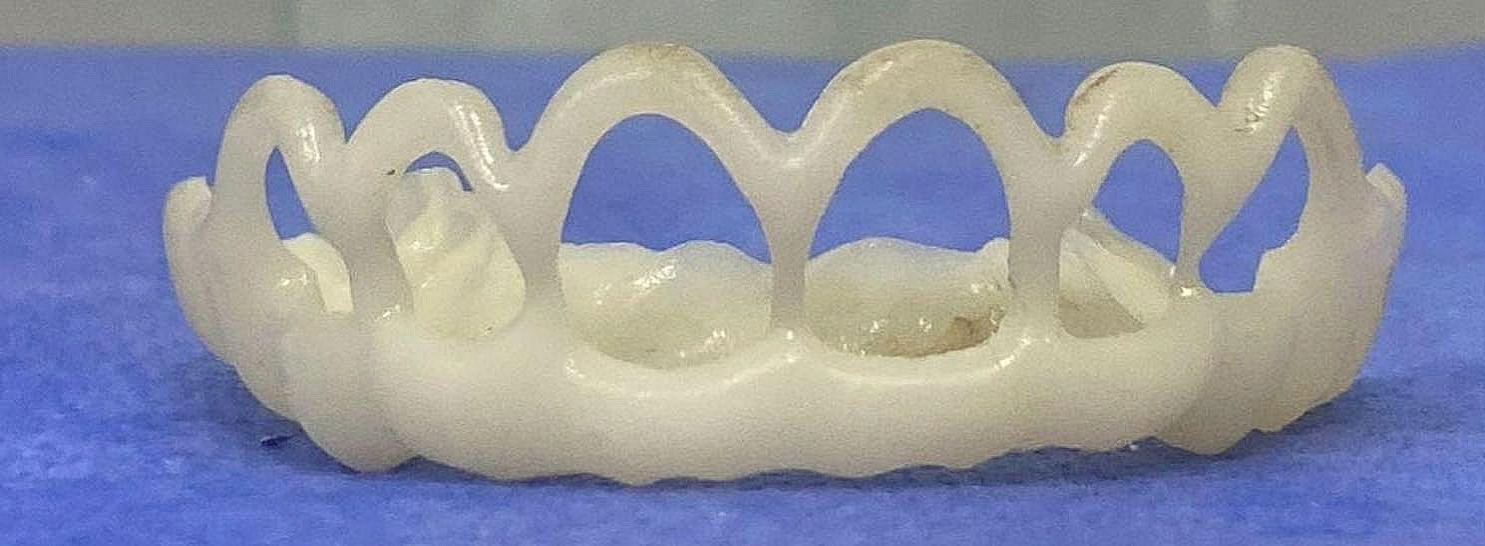
Surgical guide after printing

CBCT scans were done^3^ and were used to perform a 3D assessment of bone levels and confirm the diagnosis of APE in both groups as well as to aid in the surgical guide fabrication in the study group. A surgical guide was fabricated for patients in the study group on digital software^4^ by overlapping STL files obtained from intraoral scans and DICOM files obtained from CBCT scans, and 3D printed^5^ using PMMA (Poly methyl methacrylate) resin. The steps for the surgical guide’s fabrication are shown in Figs. (2)–(4).

### Treatment protocol

**Fig. 5:**
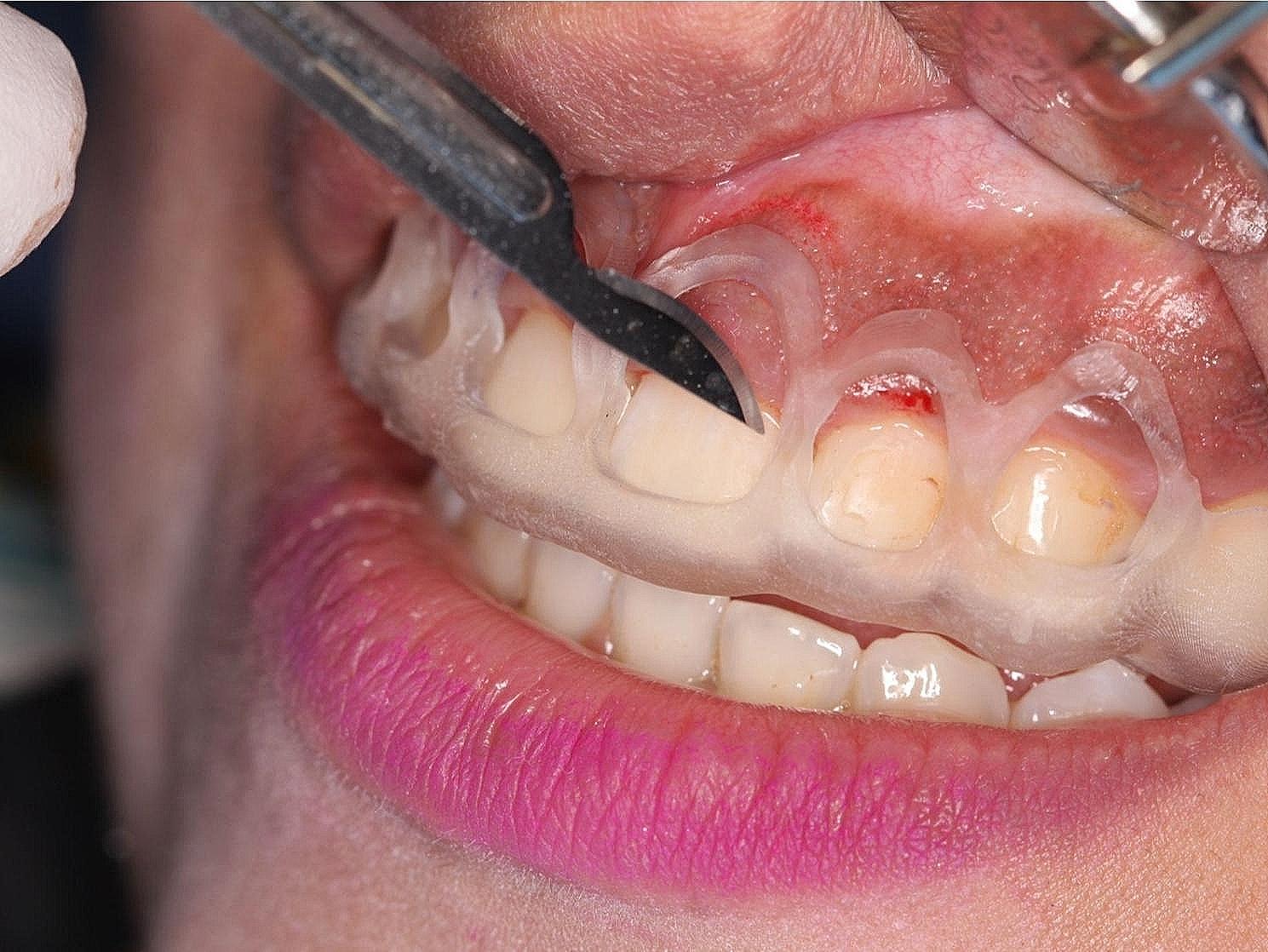
Guide placement for soft tissue incisions

**Fig. 6:**
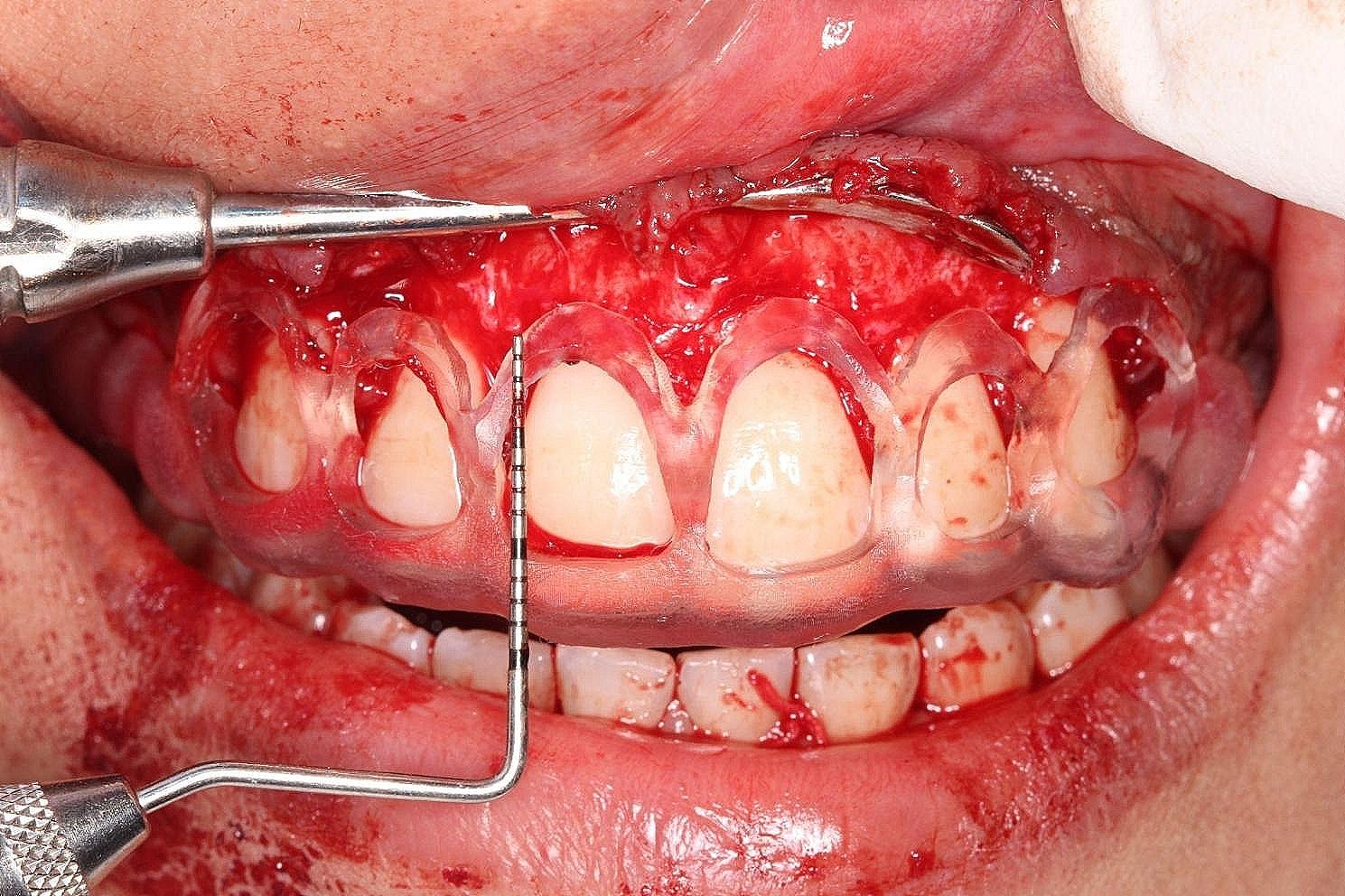
Guide placement for ostectomy

A complete full mouth non-surgical periodontal therapy (NSPT) was carried out for all patients using a DTE D5 LED Ultrasonic Scaler^6^ and universal curettes (2R − 2 L and 4R- 4 L)^7^ one week before the surgical procedure. All surgical interventions were performed after administering adequate local anesthesia^8^ (2% lignocaine hydrochloric acid with epinephrine (1:100,000)) using the infiltration technique. The control group underwent conventional ECL where the soft tissue and bone levels were removed using a 15 C blade and an end-cutting surgical bur^9^, respectively, and were measured using a graduated UNC-15 periodontal probe [12]. . The study group, however, underwent similar surgical steps except the internal bevel incisions were made according to the coronal markings on the surgical guide (Fig. (5)) and the removal of bone (ostecotomy) which was done according to the apical markings on the surgical guide (Fig. (6)). The appropriate bone levels to be removed for the new supracrestal attachment levels to form were standardized to be 3 mm apical to the CEJ in all teeth. Patients with bone exostosis in both groups underwent osteoplasty to reshape the bone. The flap was repositioned and sutured with 5–0 Polypropylene sutures in a simple interrupted manner.

Routine postoperative instructions were given to the patients in written form in which patients were instructed to consume a soft diet and cool drinks, use ice packs in the surgical area for the first 48 h (20 min on and 10 min off), avoid brushing at the surgical site for one week, and rinse twice daily with 10 mL of 0.2% chlorhexidine gluconate mouthwash^10^ for two weeks. Postoperative analgesics^11^ were prescribed to all patients thrice daily, and Antimicrobials (Amoxicillin trihydrate 500 mg thrice daily for 7 days) were prescribed for all patients. Patients were also asked to report any incidence of pain, swelling, and bleeding from the surgical site. The patients were asked to record their compliance with analgesic medication during the postoperative period. The sutures were removed after 14 days.

### Assessment

1. The operating time needed to complete the surgery was recorded on a stopwatch from the time of the first incision till the completion of bone removal.
2. The clinical features of periodontal wound healing were assessed 1 week, 3 months, and 6 months postoperatively *using* the following parameters:

*Swelling of the soft tissue (S) was evaluated visually and given a score of 0 = no swelling, 1 = moderate swelling, and 2 = pronounced swelling.

* The color of the gingiva (C), was evaluated visually and given a score of 0 = no redness, 1 = moderate redness, 2 = pronounced redness. The contra-lateral gingiva was used as a reference to judge changes in tissue color.

* Probing depth (PD), was assessed with a periodontal probe (UNC-15) with reference to the CEJ at the mesial, midfacial, and distal sites of each tooth in the operating zone. A mean of the 3 values per tooth was calculated and measurements were rounded off to the nearest millimeter.

* The bleeding index (BI) was assessed through bleeding on probing measurements using a periodontal probe and then a score was given to each individual tooth. The presence or absence of bleeding was recorded on a two-point scale (no, or within the 30s after probing).

* The plaque index (PI) was assessed using a periodontal probe. The plaque was scored on a two-point scale, as the absence or presence of plaque along the marginal gingiva.

3. Pain Scores were assessed using VAS for pain at 24 h, 7 days, and 14 days postoperatively. (Zero is for minimum pain and ten is for maximum pain).
4. Gingival margin stability (GMS) was assessed by measuring the position of the gingival margin in relation to a reference point (index with guiding grooves) and was evaluated at baseline, 3 months, and 6 months healing period.

### Statistical analysis

Power analysis was performed to have adequate power to apply statistical tests of the research hypothesis. Sample size calculation was conducted using G*Power 3.1.9.4 Software based on data from a previous study (Silva et al., 2015). The effect size was determined to be 0.80. The power of the t-test was set at 80% using a two-tailed significance level of 5% and beta level of 20%. To detect a mean difference of 0.4 mm in tissue rebound between baseline and 3 months, a sample size of 7 patients per group for a total of 14 patients will be necessary. The sample size will be increased by 15% to 8 patients per group for a total of 16 patients to compensate for dropouts.

Categorical data were presented as frequency and percentage values and were analyzed using the chi-square test for intergroup comparisons and Cochran’s Q test for intragroup comparisons followed by pairwise comparisons utilizing multiple McNemar’s test with Bonferroni correction. Numerical data were presented as mean and standard deviation values. They were analyzed for normality using the Shapiro-Wilk test. Operating time, gingival margin stability, and probing depth data were normally distributed and were analyzed using independent t-test for intergroup comparisons and repeated measures ANOVA followed by Bonferroni post hoc test for intragroup comparisons. Other numerical data were not normally distributed and were analyzed using the Mann-Whitney U test for intergroup comparisons and Friedman’s test followed by the Nemenyi post hoc test for intragroup comparisons. The significance level was set at *p* ≤ 0.05 within all tests. Statistical analysis was performed with R statistical analysis software version 4.1.3 for Windows^12^.

## Results

A total of 16 patients aged 21–30 years were enrolled in the study. Statistical analysis of the control group’s demographic data revealed an age range (21–28) with a gender distribution of 6 females and 2 males. The study group’s demographic data revealed an age range (21–30) with a gender distribution of 7 females and 1 male. Demographic data comparison between both groups didn’t reveal a statistically significant difference.

The changes in the mean values ± standard deviations of Swelling (S), Color (C), PD, Pain levels, Gingival margin stability (GMS), and Operating time indices are shown in Table (1) The changes in the frequency and percentage of Bleeding Index (BI) and PI are shown in Table (2).

The results of the present study showed that there was no statistically significant difference in regard to S or C at the different intervals of the study between both groups. There was a statistically significant difference between 1 week and 3 months as well as 1 week and 6 months within each group. Regarding the PD (that was not assessed at the 1 week follow up period due to the incomplete formation of the junctional epithelium) among the two groups at the different intervals of the study, there was no statistically significant difference. The results of the BI and PI showed that the highest percentage of bleeding was at 1 week with a score of 100% in both groups. This decreased in the 3-month follow-up period and further decreased in the 6-month follow-up period. These results collectively determined the wound healing score.

**Table 1:** The changes in the mean values ±standard deviations of Swelling (S), Color (C), Probing Depth (PD), Pain levels, Gingival margin stability (GMS) and Operating time

		Time Interval						
		Baseline	24 h	1 week	2 weeks	3 months	6 months	p-value
Swelling score (Mean±SD)	**Control group**			1.25±0.46 A		0.25±0.46B	0.12±0.35B	0.002*
	**Study group**			1.25±0.46 A		0.25±0.46B	0.00±0.00B	<0.001*
	**p-value**			1ns		1ns	0.382ns	
Color score (Mean±SD)	**Control group**			1.12±0.64 A		0.50±0.53B	0.25±0.46B	0.009*
	**Study group**			1.38±0.52 A		0.50±0.53B	0.12±0.35B	0.005*
	**p-value**			0.460ns		1ns	0.587ns	
Probing depth (mm) (Mean±SD)	**Control group**	2.85±0.23 A				2.53±0.32 A	2.49±0.44 A	0.101ns
	**Study group**	2.86±0.27 A				2.41±0.26 A	2.31±0.16 A	0.219ns
	**p-value**	0.117ns				0.456ns	0.312ns	
Pain levels	**Control group**		5.12±0.64 A	2.75±1.98AB	0.88±0.83B			0.001*
	**Study group**		4.00±1.41 A	1.12±0.83AB	0.25±0.46B			<0.001*
	**p-value**		0.114ns	0.100ns	0.111ns			
Operating time	**Control group**	53.88±2.42						
	**Study group**	44.62±2.33						
	**p-value**	<0.001*						
Gingival margin stability (mm) (Mean±SD)	**Control group**	7.15±0.39B				8.68±0.44 A	8.46±0.44 A	<0.001*
	**Study group**	7.12±0.39B				8.68±0.44 A	8.46±0.44 A	<0.001*
	**p-value**	0.900ns				1ns	1ns	

**Table 2:** The changes in the frequency and percentage of Bleeding Index (BI) and Plaque Index (PI)

	Time Interval												
	1 week				3 months				6 months				p-value
Bleeding index	**No**		**Yes**		**No**		**Yes**		**No**		**Yes**		
	n	%	n	%	n	%	n	%	n	%	n	%	
Control group	0 A	0%	8	100%	6B	75%	2	25%	6B	75%	2	25%	0.006*
Study group	0 A	0%	8	100%	4AB	50%	4	50%	7B	88%	1	13%	0.010*
odds ratio (95%CI)	NA				3 (0.36:24.92)				0.43 (0.03:5.99)				
p-value	NA				0.302ns				0.522ns				
Plaque index	**No**		**Yes**		**No**		**Yes**		**No**		**Yes**		
	n	%	n	%	n	%	n	%	n	%	n	%	
Control group	0 A	0%	8	100%	6B	75%	2	25%	6B	75%	2	25%	0.006*
Study group	0 A	0%	8	100%	4AB	50%	4	50%	6B	75%	2	25%	0.018*
odds ratio (95%CI)	NA				3 (0.36:24.92)				1 (0.1:9.61)				
p-value	NA				0.302ns				1ns				

There was no statistically significant difference between the two groups regarding pain levels during the different time frames of the follow-up period. However, there was a statistically significant difference between 1 day and 1 week as well as 1 day and 14 days in the control group. The same applies to the study group.

When it came to the operating time, there was a statistically significant difference between the control and study groups with the study group being significantly lower.

Finally, the gingival margin stability that was measured by measuring the clinical crown length showed no statistically significant difference between the two groups during the different time frames of the follow-up period. However, there was a statistically significant difference between baseline and 3 months as well as baseline and 6 months in the conventional ECL group. The same applies to the digitally assisted ECL group. There was no statistical significance between 3 months and 6 months in either group.

## Discussion

Modern dentistry aims to satisfy patients’ expectations and dental esthetic requirements while also ensuring that health and function are maintained. Excessive gingival exposure might affect a patient’s smile’s esthetics. One of the main causes of EGD is APE in which the gingival margin and alveolar bone levels cause atypical clinical crown dimensions. Since the increased demand for gummy smile correction skyrocketed, authors suggested the use of surgical ECL [13].

ECL to treat APE requires the removal of soft tissue as well as alveolar bone recontouring using manual instruments. This includes the use of periodontal probes or Chu gauges to determine the new soft tissue and bone levels. The amount of ostectomy has been a topic for debate amongst many authors. Some suggested the use of the margin of the flap as a reference instead of the CEJ. More recently, others advocated that the bone level should vary according to the case with at least a 2 mm distance from the bone crest to the CEJ being adequate in most cases [14, 15].

Bone-sounding and periapical radiograph examinations are frequently carried out to identify the bone crest. These approaches, however, could be difficult to use and might result in unreliable evaluations. In cases where maxillary anterior teeth exhibit incisal wear followed by compensatory eruption, the CEJ is more coronal than predicted. If the surgical guide is constructed based on only a diagnostic wax-up, performing ECL on such cases may result in excessive removal of soft tissues, which could lead to root exposure and necessitate a restorative procedure that was not initially planned. To account for patient variability and lessen the possibility of under or over-contouring hard and soft tissues, a precise outline of the patient’s anatomical CEJ site needs to be done. This is facilitated by CBCT. CBCT has been suggested as a precise and reliable alternate way for identifying APE [15].

Over time, new techniques were introduced to the conventional ECL procedure. Among these techniques were vacuum-formed or acrylic resin surgical guides. The new clinical crown length is often determined by diagnostic waxing to create these guides; however, manual measurements are used in this waxing technique to determine the required alveolar bone crest level. The periodontal phenotype and site-specific factors including buccal bone thickness, gingival recession, root structure, and tooth shape are different variables that compromise the accuracy of these measurements. Hence, this technique may not be accurate for patients who require bone resection, leading to unanticipated aesthetics after therapy [9].

Newer technologies were introduced to decrease these inaccuracies. One of these techniques is the Computer-aided design and computer-aided manufacturing (CAD-CAM) technologies that have transformed surgical planning in dentistry. A 3D virtual patient can be made to noninvasively mimic a complete treatment by combining hard and soft tissue imaging data. However, there aren’t many digital workflows for crown-lengthening procedures. Mendoza- Azpur et al., 2020 outlined a digital process for a surgical crown-lengthening technique that used a single digitally designed, surgical guide [16]. This process was able to compare the limitations of the soft and hard tissues to determine whether alveoloplasty is necessary.

Another technology that was introduced is 3D printing of the surgical guide. The guide is fabricated based on CBCT measurements and an intra-oral scan. This can help with ECL when no prosthetic therapy is required. Instead of using diagnostic waxing to fabricate the guide, this approach uses the tooth’s existing anatomy to produce predictable results [15].

According to some researchers, using a surgical guide during implant placement is more accurate than using alternative techniques [17]. Others have stated that despite dental implants’ excellent precision when performed with a surgical guide, accuracy from free-hand implant surgery has been adequate and acceptable for the majority of clinical situations. Studies found that using surgical guidance reduced implant failure rates by almost threefold. The duration of surgery, pain intensity, and analgesia, as well as cases of trismus and bleeding, were all found to be significantly lower in flapless patients than in patients with free-hand surgery [18].

Studies involving dual guides for ECL concluded that using the guides proved to be more accurate when it came to determining the soft tissue and bone levels to be removed, especially when it comes to inexperienced surgeons. This is due to the difficulty in determining the position of the gingival level after the flap is elevated when performing free-hand surgery. On the contrary, dual guides provide predetermined bone levels that facilitate the ostectomy procedure to be more precise [19]. This is an important clinical consideration for the treatment since gingival recession might be caused by excessive bone removal. Moreover, coronal gingival margin regrowth after ECL and partial remission of APE were linked to inadequate bone excision [20].

The findings of several randomized controlled trials also demonstrated that guided surgery implants are more expensive than free-hand surgery implants but provide greater accuracy, less pain and swelling, and shorter surgery times. decreasing the invasion while also speeding up the procedure [21–23].

Since the periodontal phenotype has an important effect on the stability of the gingival margin, it is crucial to maintain distances of 2 and 3 mm, respectively, between the CEJ and the alveolar crest in patients with thin and thick phenotypes. This is because when compared to thin phenotypes, thick phenotypes appeared to be associated with a greater GM rebound following an ECL procedure [5]. Visual inspection should not be considered a credible method for assessing the periodontal phenotype; instead, CBCT should be employed [24].

As a result, 3D-printed surgical guides were chosen in this study as an aid to surgical ECL. The subjects of the study are diagnosed with APE as well as thick gingival phenotype with the use of CBCT.

The follow-up period of this study was determined according to Ong et al., 2011 who stated that if a buccal flap was raised and the bone was exposed, it would take eight to twelve weeks for the tissue to mature and stabilize [17]. For many patients, six months of healing period is advised if significant bone reduction (2-3 mm) is required for the ECL.

The parameters that were assessed during this study were: wound healing score (swelling, color, PD, BI, PI) according to Hagenaars et al., 2004 [25], operating time, pain levels, and GMS according to Dominguez et al., 2020 [5].

The results that were achieved in terms of the swelling and color may be due to the healing of the soft tissue in the first week as well as the patient’s inability to perform adequate oral hygiene measures. However, those regarding the probing depth may be due to the inclusion criteria of the patients with no periodontal disease (at baseline) and the normal suprarenal attachment formation during healing at 3 months and 6 months in both groups. These results are contrary to those of Lanning et al., 2003 who found a statistically significant difference between the PD at baseline and the 3 months as well as the 6-month follow-up period [6]. The results of the BI and PI on the other hand may be due to the completion of soft tissue healing as well as the ability of the patient to perform oral hygiene measures more comfortably as the soft tissue healed. The results of the previous criteria (under the parameter ‘Wound healing’) were in accordance with Colombo et al., 2017 who stated that wound healing was not affected by the use of surgical guides during implant placement. The clinical parameters were similar in both groups of his study [26]. 

When it came to the pain levels, the results achieved might have been due to the healing of the soft tissue reducing the patient’s discomfort. These results were similar to that of Hamzani et al., 2016 who correlated patient satisfaction with wound healing in different Oral and Maxillofacial surgeries [27]. Since wound healing was statistically similar in both groups, it was expected that the patient satisfaction levels would be similar as well. However, the results were in contrast to Colombo et al., 2017 who stated that patient satisfaction was increased in groups that were operated on using surgical guides during implant placement [26].

Regarding the operating time, the results achieved may have been due to the lack of need to manually measure soft tissue as well as bone levels during every surgical step. This coincides with the findings of Colombo et al., 2017 who concluded that the operating time was significantly reduced when using a surgical guide during implant placement [26], and Ballard et al., 2020 who stated that the operation time decreases due to the use of surgical guides in orthopedic and maxillofacial surgery [28, 29].

Finally, the gingival margin stability’s results may be due to the stabilization of the new gingival margin levels after the healing period. These results are in agreement with those of Dominguez et al., 2020 and Aroni et al., 2019 who found no statistically significant difference between the clinical crown length after ECL during the follow-up periods of 3 months and 6 months [5],[30]. These results are similar to those of Carrera et al., 2022 where there was no statistically significant difference between the gingival margin stability in the group receiving freehand ECL vs. dual guide-assisted ECL [19].

This study revealed that the use of 3D-printed surgical guides in the treatment of EGD gave better clinical results in terms of operating time than conventional surgical ECL These results were in accordance with most of the literature as the use of surgical guides removes the time required to manually measure soft tissue and bone levels before and after every step.

Also, this study showed that the use of 3D printed surgical guides failed to surpass the wound healing scores, the stability of GM after the surgical procedure as well as the patient satisfaction scores of the conventional surgical ECL and this is why conventional surgical ECL is considered the gold standard method in treatment of EGD due to APE.

## Conclusion

Within the limitations of this study, it can be concluded that:

1. Digitally assisted surgical ECL helps shorten the operating time and reduces the possibility of human errors during the measurements. This will be useful in helping practitioners (who might have a higher possibility for errors) to achieve better results.
2. Better results in terms of operating time were achieved by the digitally assisted ECL Group.
3. Similar results in terms of wound healing, patient satisfaction, and GMS were found in both groups.

### Electronic supplementary material

Below is the link to the electronic supplementary material.

Supplementary Material 1

Supplementary Material 2

## Data Availability

The datasets used in the current study are available from the corresponding author upon request.

## Footnotes

1 www.randomization.org.

2 Hu-Friedy Mfg. Co., LLC, USA.

3 Soredex Cranex 3D Dental Imaging System, Finland.

4 Blenderfordental headquarters are at Southport, Queensland, Australia.

5 PHROZEN TECH CO., LTD.287, NIUPU RD., XIANGSHAN DIST., HSINCHU CITY 30,091, Taiwan.

6 Guilin Woodpecker Medical Instrument Co., LTD. China.

7 Nordent Manufacturing Inc. | 610 Bonnie Lane | Elk Grove Village, IL 60,007 (Nordent, USA).

8 Artinibsa 2% Inibsa Dental S.L.U, Ctra. Sabadell a Granollers, KM 14,5 (C-155), 08185 Lliçà de Vall (Barcelona), Spain.

9 Dentsply Sirona, USA.

10 Hexitol; Kahera Pharmaceutical, Cairo, Egypt.

11 Brufen 400 mg; Kahira Pharm. & Chem. Ind. Co., Under license from: Abbott Laboratories.

12 ((R Core Team (2022). R: A language and environment for statistical computing. R Foundation for Statistical Computing, Vienna, Austria. URLhttps://www.R-project.org/)).

## Abbreviations

EGD: Excessive Gingival Display
APE: Altered Passive Eruption
CEJ: Cemento Enamel Junction
CBCT: Cone Beam Computed Tomography
STL: Standard Tessellation Language
DICOM: Digital Imaging and Communications in Medicine
ECL: Esthetic Crown Lengthening
CAD-CAM: Computer-Aided Design and Computer-Aided Manufacturing
S: Swelling
C: Color
CAL: Clinical attachment level
PD: Probing Depth
PI: Plaque Index
BI: Bleeding Index
GMS: Gingival Margin Stability
