# Supplementary material for: Treatment of excessive gingival display using conventional esthetic crown lengthening versus computer guided esthetic crown lengthening: (a randomized clinical trial)
Source: BMC Oral Health. 2024 Mar 9;24:317. doi: 10.1186/s12903-024-04080-5 (PMC10925018; doi:10.1186/s12903-024-04080-5)
Supplement: Supplementary file 2 — Supplementary Material 2 [file 12903_2024_4080_MOESM2_ESM.docx]

**Procedure steps**

**(Preoperative, intraoperative, postoperative, and follow up)**

| Preoperative clinical photograph | 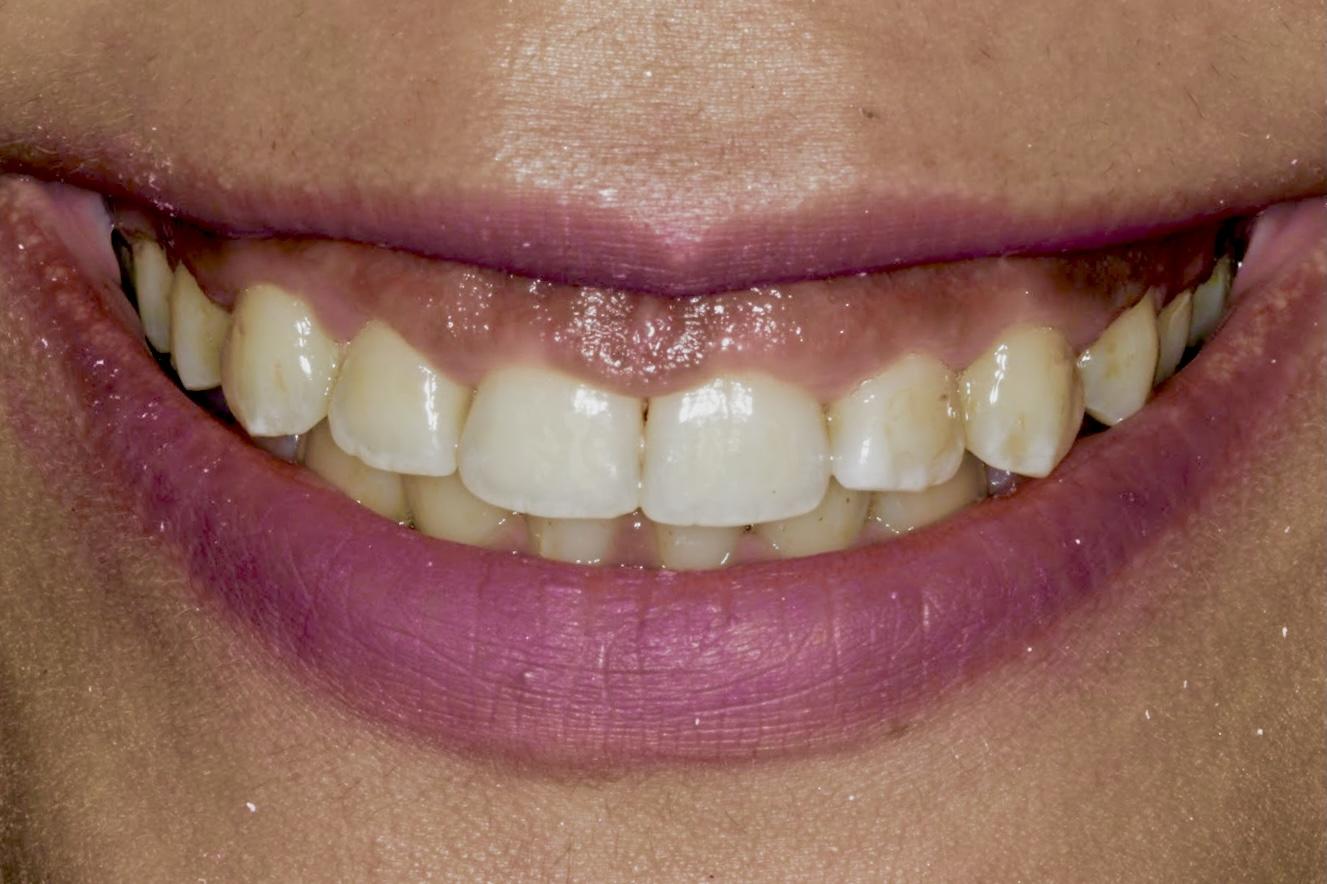 |
| --- | --- |

**Group**

| Clinical picture showing guide adaptation. | 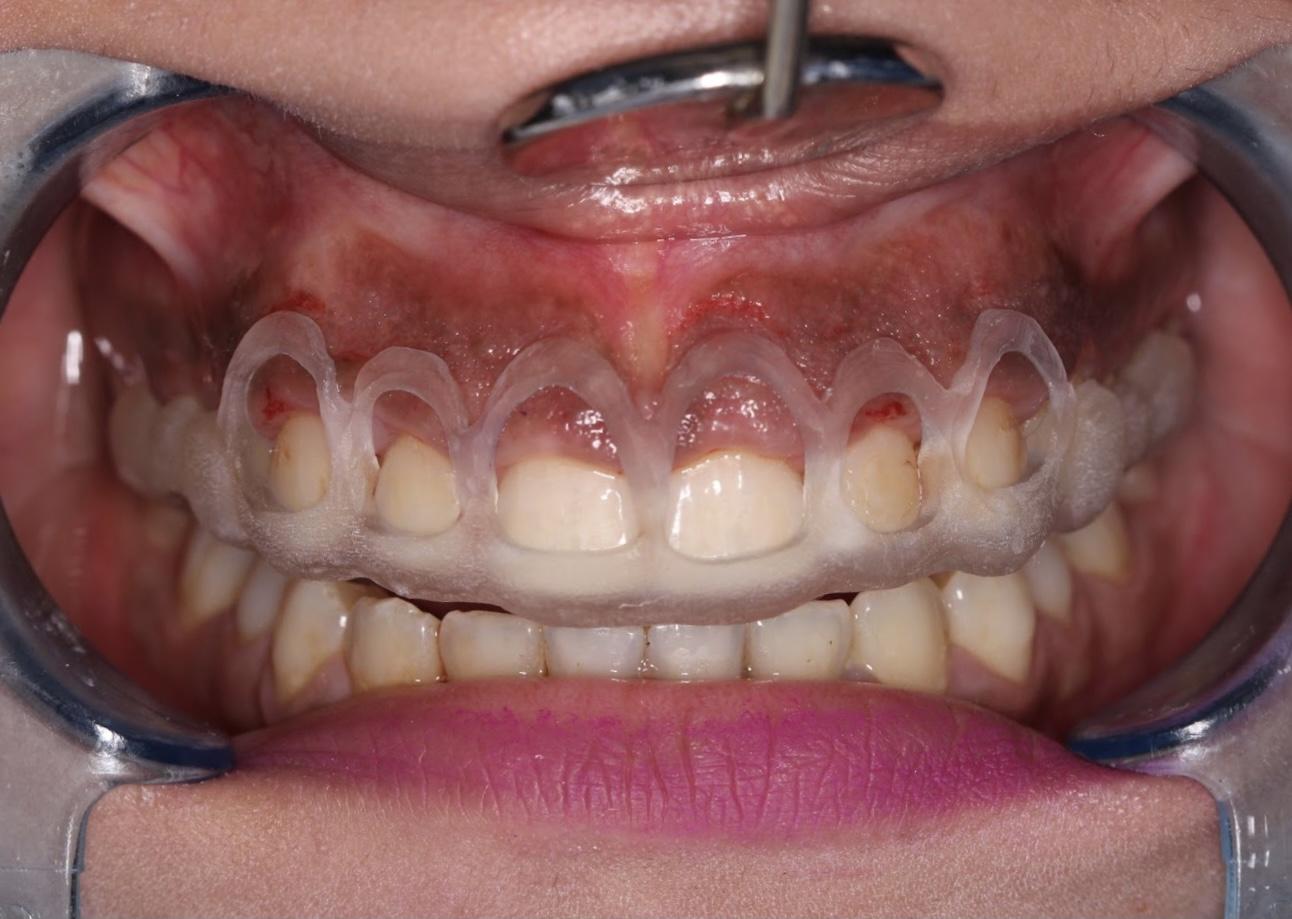 |
| --- | --- |
| Clinical picture showing internal bevel incisions that were followed by sulcular incisions. | 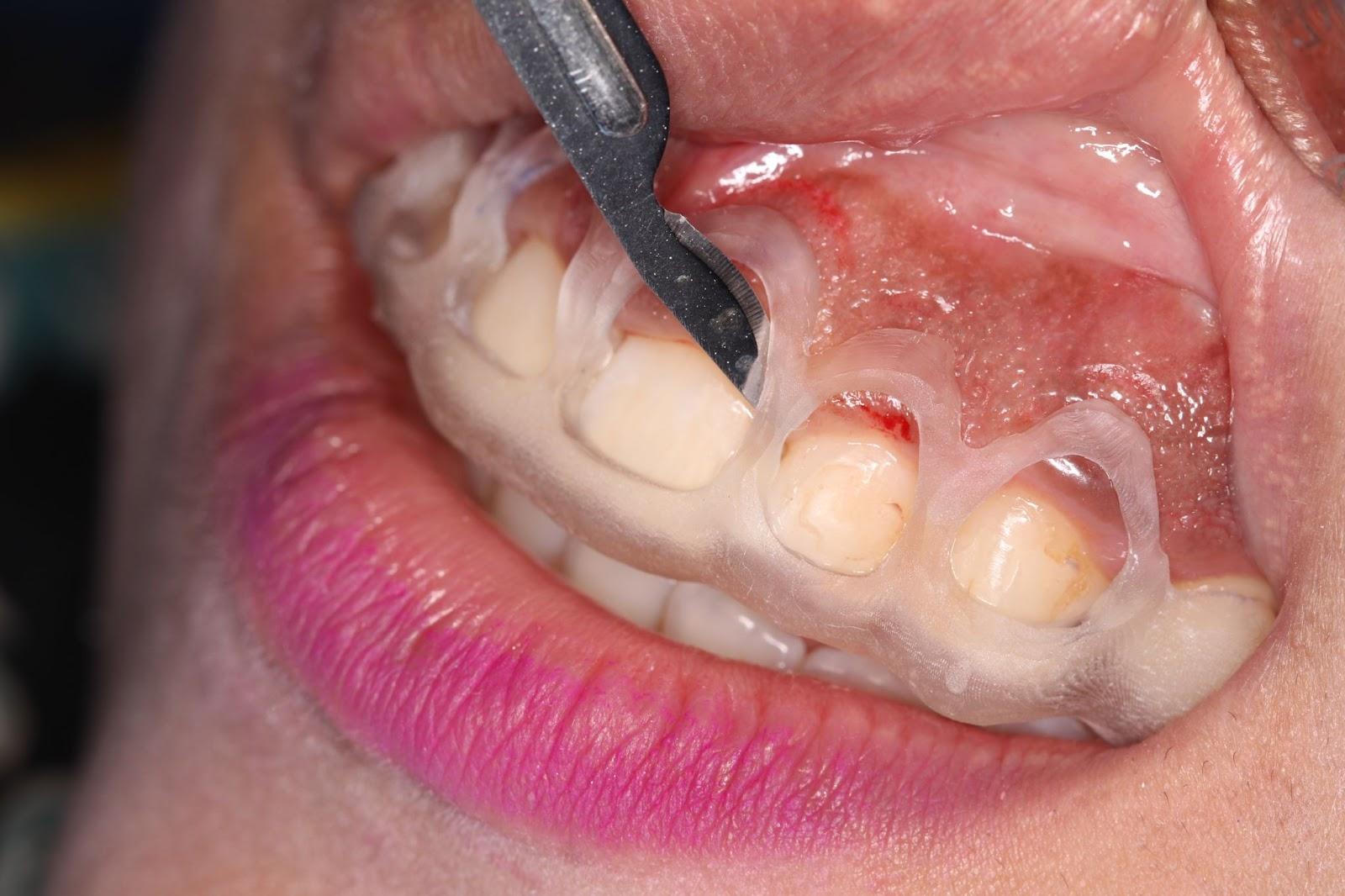 |

**Study Group**

| Clinical picture showing bone levels that require removal after surgical guide adaptation. | 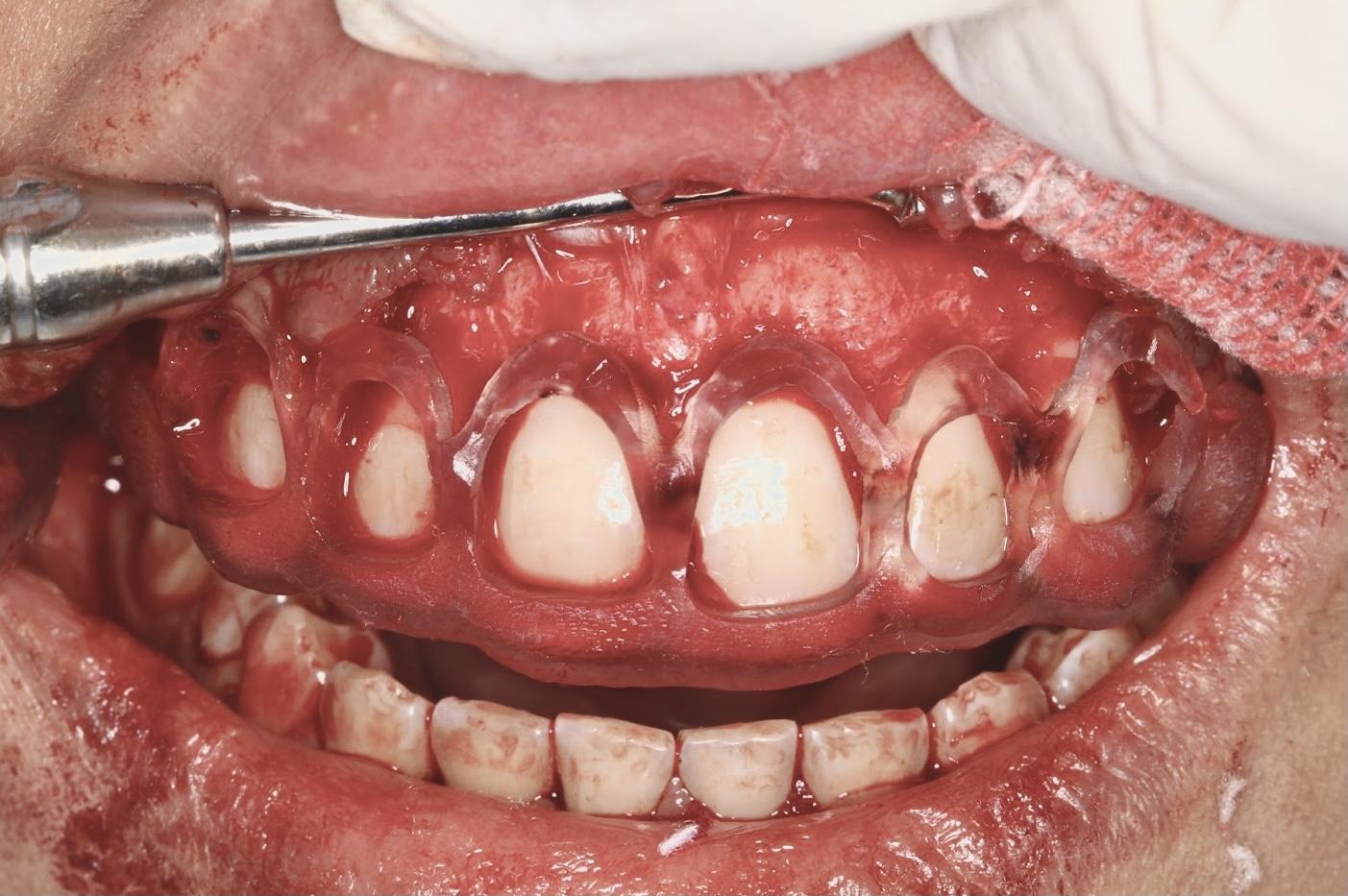 |
| --- | --- |

**Study Group**

| Clinical picture showing 6 months follow up | 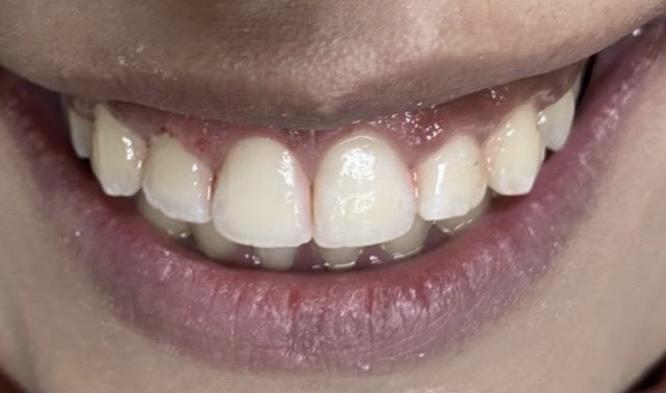 |
| --- | --- |
